# Management of Esophageal Squamous Cell Carcinoma With Esophageal Stent Placement in an Elderly Patient With Dysphagia

**DOI:** 10.7759/cureus.50483

**Published:** 2023-12-13

**Authors:** Charles Vallejo, Yousra Gheit, Jerry Qi, Talwinder K Nagi, Zoilo K Suarez, Muhammad A Haider, Touqir Zahra

**Affiliations:** 1 Internal Medicine, Florida Atlantic University Charles E. Schmidt College of Medicine, Boca Raton, USA

**Keywords:** dysphagia in the elderly, esophageal cancer (ec), esophageal adenocarcinoma, esophageal squamous cell carcinoma (scc), esophageal stent

## Abstract

Esophageal cancer is typically identified as squamous cell carcinoma or adenocarcinoma. There are multiple risk factors that may contribute to esophageal squamous cell carcinoma including smoking, alcohol consumption, and the human papillomavirus. Lesions may appear ulcerated, friable, and circumferential and may obstruct the esophagus. Therefore, patients may complain of non-specific symptoms including dysphagia, weight loss, and retrosternal discomfort. Clinicians often rely on an upper endoscopy with biopsy to confirm the diagnosis. Computed tomography scans and endoscopic ultrasounds are also employed to assess the extent of malignant spread. Management may involve endoscopic resection for superficial lesions or surgical resection for lesions penetrating the submucosa. Esophageal stents may play a role, specifically as a palliative measure for enhancing oral intake. We present an instance of utilizing a self-expandable, metal-covered esophageal stent with balloon dilation in the setting of a newly diagnosed esophageal squamous cell carcinoma lesion in a 73-year-old female. Ultimately, the use of an esophageal stent in this patient helped improve the patient's oral intake during her course of hospitalization. Her diet was slowly advanced to clear liquids and progressively to a low-residue diet before being discharged to follow-up with her diagnosis as outpatient with gastroenterology.

## Introduction

Esophageal cancer is known to be a deadly malignancy and represents the fifth most common gastrointestinal cancer in the United States with approximately 16,940 new cases per year [1]. Histologically, esophageal cancers are mainly identified as squamous cell carcinoma (SCC) or adenocarcinoma (ACA). There are many risk factors for SCC including smoking, alcohol consumption, a diet that is low in fruits and vegetables, as well as drinking beverages at high temperatures. Notably, human papillomavirus (HPV) has also been correlated with an increased incidence of SCC in the upper esophagus. However, in general, the United States is considered a low-risk area for esophageal SCC and instead has been witnessing an increase in incidence of esophageal ACA. This may be due to the upsurge of obesity and gastroesophageal reflux disease (GERD). Conversely, SCC is mainly found in the "esophageal cancer belt" of northern Iran, southern Russia, central Asian countries, and northern China [1].

Initially, early lesions may be subtle, but more advanced lesions may be ulcerated and circumferential and infiltrate the submucosa. Spread may occur via the lymphatic system to regional lymph nodes. Distant metastases may subsequently involve the liver, lung, and bone marrow. Patients typically present with complaints of dysphagia, substantial weight loss, and other non-specific symptoms such as retrosternal discomfort or burning sensation. To evaluate, clinicians may start with a barium swallow, but an upper endoscopy with biopsy is performed to confirm the diagnosis. Computed tomography (CT) scans of the thorax and abdomen may be performed to determine the extent of the primary tumor as well as look for any potential liver metastasis and celiac lymphadenopathy. However, it should be noted that endoscopic ultrasound (EUS) has now become the standard for locoregional staging and for assessing tumor depth and mediastinal lymph node involvement. It also allows a fine-needle aspiration biopsy of lymph nodes. Management typically involves either endoscopic resection for superficial, limited mucosa disease or surgical resection for lesions penetrating the submucosa. Neoadjuvant chemoradiation of resectable lesions and palliative systemic therapy for unresectable or metastatic disease also play a role [1]. We present a case of newly diagnosed esophageal SCC requiring an esophageal stent to improve per os (PO) intake.

## Case presentation

**Figure 1:**
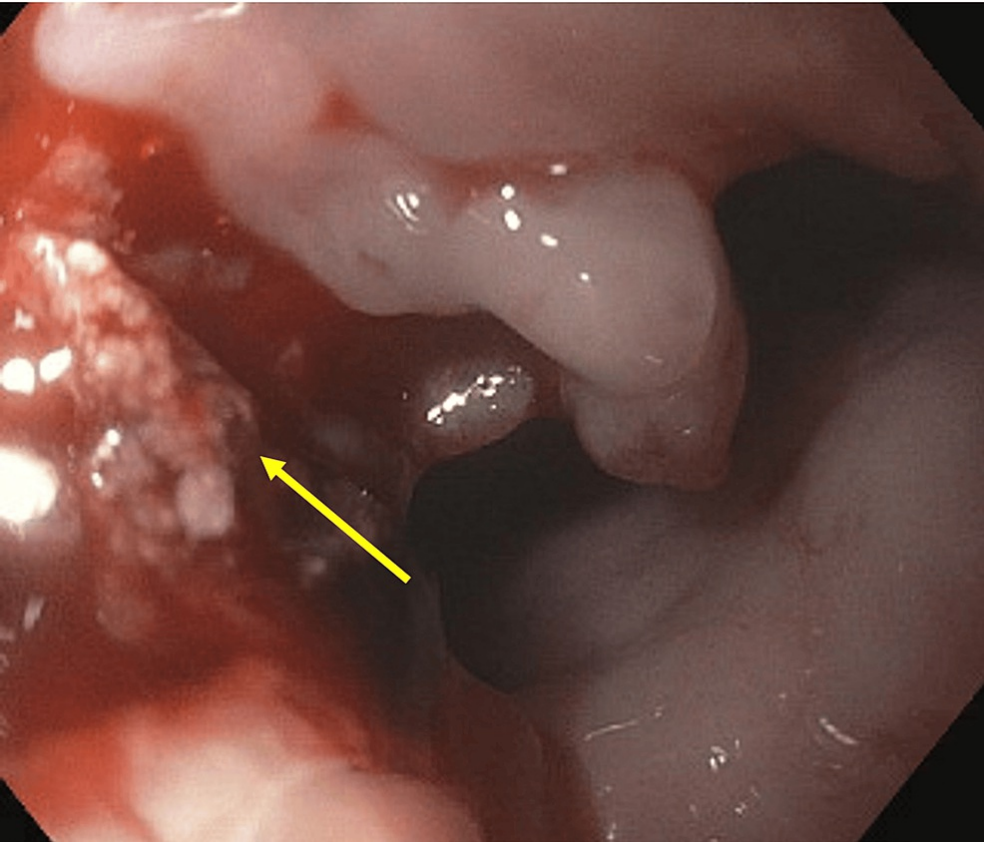
EGD depicting a nearly obstructing, friable, ulcerated mass extending from the proximal to mid-esophagus. EGD: esophagogastroduodenoscopy

A 73-year-old female with a past medical history of chronic obstructive pulmonary disease (COPD) and hypertension presented to the emergency department due to symptoms of exacerbated dysphagia for the past three months. She endorsed dysphagia to solids initially, but progressively complained of dysphagia to liquids as well. She reported a 10-pound weight loss since the initiation of her symptoms. She was admitted and was made nil per os (NPO) upon admission due to inability to tolerate both solids and liquids. The patient was taken for an esophagogastroduodenoscopy (EGD) the following day. The EGD revealed a nearly obstructing, friable, ulcerated mass extending from the proximal to mid-esophagus, approximately spanning from 27 to 34 centimeters down the length of the esophagus as seen in Figure 1. The pathology report revealed that the mass was an infiltrating, poorly differentiated, SCC of the esophagus. The pathology report was also negative for HPV testing.

**Figure 2:**
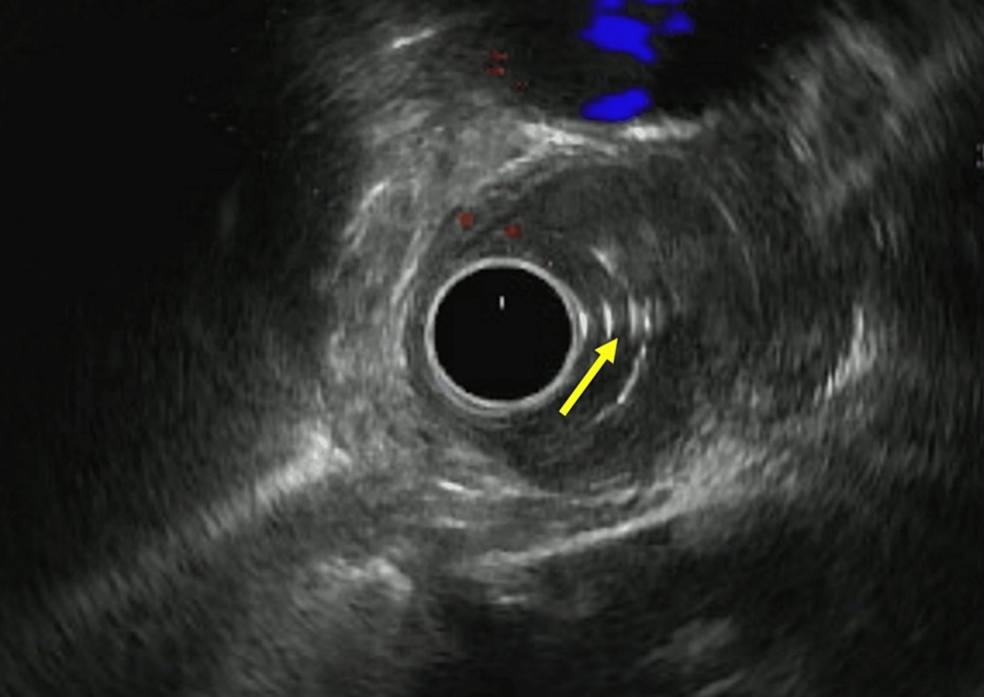
EUS depicting esophageal SCC classified as stage 3: Tumor-3 (T3) and Nodes-2 (N2). EUS: endoscopic ultrasound; SCC: squamous cell carcinoma

**Figure 3:**
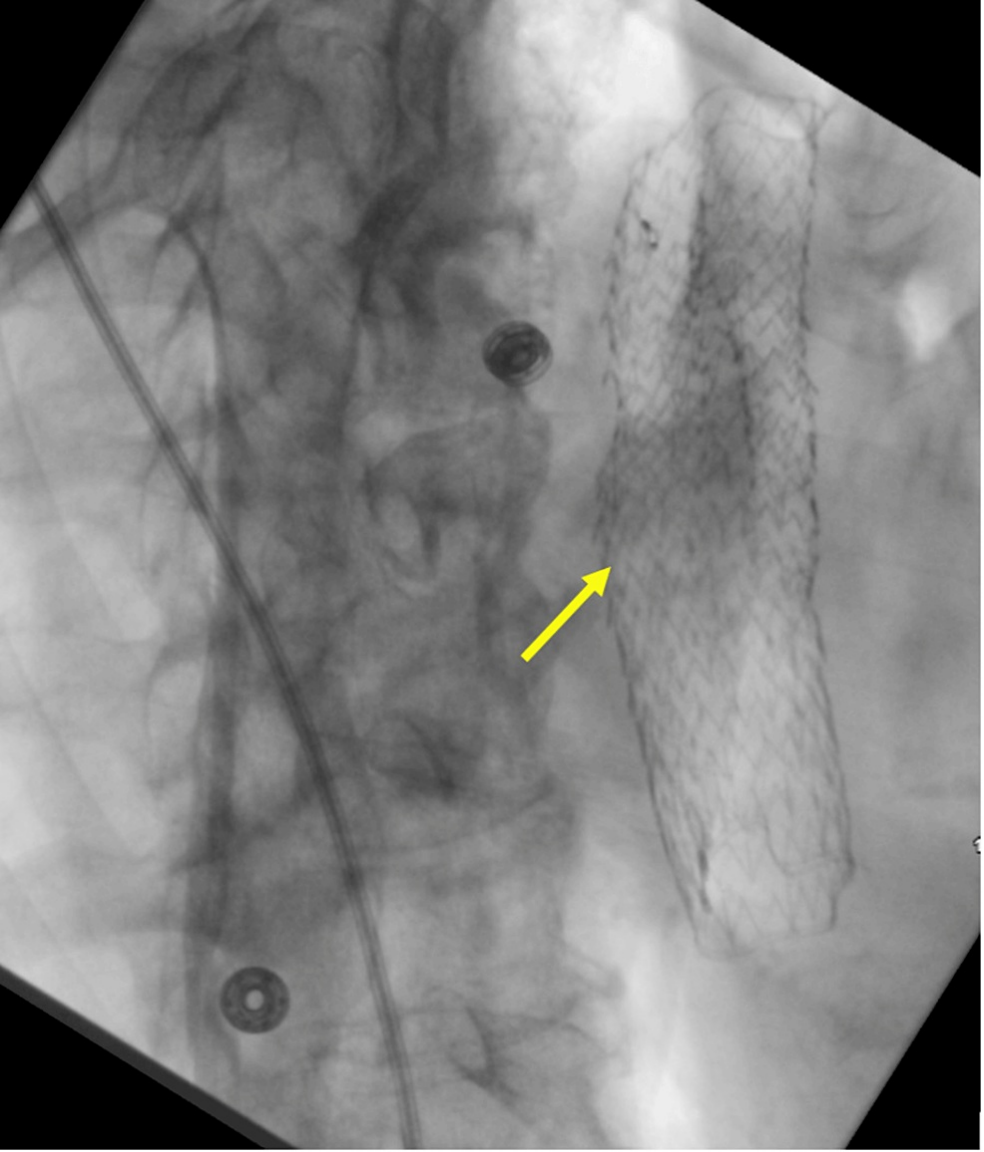
A 120 millimeter x 23 millimeter self-expanding covered metal esophageal stent was deployed, with balloon dilation of the proximal opening.

Further evaluation with CT scans of the chest and abdomen and pelvis revealed no distant metastases. Subsequently, a decision was made to perform an EUS with concomitant esophageal stent placement for staging and palliative feeding purposes. The EUS revealed esophageal SCC classified as stage 3: Tumor-3 (T3) and Nodes-2 (N2) as seen in Figure 2. A 120 millimeter x 23 millimeter self-expanding covered metal esophageal stent was deployed, with balloon dilation of the proximal opening as shown in Figure 3. The patient tolerated the procedure well and departed the endoscopy suite in stable condition and returned back to the medical floors. During the remainder of her hospitalization, her diet was slowly advanced to clear liquids and was eventually transitioned to a low-residue diet. She was discharged from the hospital on day 6 of her admission and advised to follow-up with her new diagnosis as outpatient with gastroenterology.

## Discussion

Dysphagia is typically the predominant symptom in patients with SCC of the esophagus and often leads to weight loss and malnutrition [2]. Because esophageal cancer is often first diagnosed at an advanced stage, a great number of patients require some form of palliative treatment to relieve the esophageal stricture, maintain caloric intake, and improve the quality of life. Among various treatment options for malignant dysphagia, endoscopically inserted stent is the most widely used method today. The first stents used were rigid plastic stents that were effective but accompanied complications such as perforation, migration, and food impaction. Self-expanding metal stents (SEMSs) were then developed in the 1990s and provided immediate dysphagia palliation in more than 85% of patients. They were also associated with significantly reduced stent-related mortality, perforation, and migration compared to plastic stents [2]. An array of self-expanding stents have since materialized to reduce tumor in-growth through the open mesh and minimize stent migration. Currently available options include uncovered SEMSs, partially covered SEMSs, fully covered SEMSs, and self-expandable plastic stents (SEPSs) [3]. Partially covered SEMSs have been the most frequently used stent clinically worldwide; however, stent selection should be tailored to the individual patient's characteristics [2-4]. In addition to patients with unresectable esophageal carcinoma, placement of SEMSs is also indicated for patients on neoadjuvant therapy awaiting definitive surgical treatments [2].

While SEMSs are widely used for palliation of dysphagia, there is no standardized optimal method, and other modalities exist [2]. A randomized trial comparing the efficacy of brachytherapy versus partially covered SEMS placement found brachytherapy to have a more latent onset of dysphagia improvement and a superior long-term dysphagia relief after three months of follow-up. Brachytherapy was also associated with fewer major complications compared to SEMSs. However, limited availability of brachytherapy and the uncertainty of optimal dose schedule hinder it from widespread use [2-3]. While endoscopic ablation methods such as photodynamic therapy (PDT) and laser therapy provided comparable palliation to SEMS, meta-analysis concluded ablative therapies required more re-interventions [2-3].

In our case, these factors were considered when placing a self-expanding covered metal esophageal stent for dysphagia palliation. The patient's improved PO intake after stent placement supports esophageal stent as a first-line modality for immediate malignant dysphagia relief. Treatment for patients with severe dysphagia in the setting of esophageal SCC should center around improving dysphagia symptoms and maintaining adequate caloric intake while definitive treatment options are determined.

## Conclusions

Esophageal SCC should be suspected in the setting of non-specific symptoms such as dysphagia, weight loss, and retrosternal discomfort. Typically, treatment options include esophageal or surgical resection along with neoadjuvant chemoradiation of resectable lesions. However, the placement of an esophageal stent may be considered to provide patients with palliative treatment. Stents may relieve the esophageal stricture, maintain caloric intake, and improve the quality of life.
